# Photocatalytic Microbial Fuel Cells and Performance Applications: A Review

**DOI:** 10.3389/fchem.2022.953434

**Published:** 2022-06-30

**Authors:** Yao Tong, Julong Wei, Rick Mo, Hailing Ma, Fujin Ai

**Affiliations:** ^1^ Hoffmann Institute of Advanced Materials, Shenzhen Polytechnic, Shenzhen, China; ^2^ School of Mechanical Engineering, Shandong University, Jinan, China; ^3^ Hong Kong Productivity Council(HKPC), Hong Kong, China; ^4^ College of Health Science and Environmental Engineering, Shenzhen Technology University, Shenzhen, China

**Keywords:** microbial fuel cell, photocatalysis, photocathode, energy production, degradation

## Abstract

In recent years, photocatalytic microbial fuel cells have gradually become a hot research topic in pollutant treatment, using either *in situ* or indirectly the oxidation of organic pollutants by catalytic materials under light and the biodegradation and mineralization of various components in wastewater by microorganisms, or through the generation of electricity by the microbial fuel cell (MFC) system to promote the photogeneration and separation of electrons and holes by the catalytic materials of the photocatalytic cell (PC) system. This study aims to provide new ideas for the development of environmentally friendly wastewater treatment technologies by investigating the use of photocatalytic cells for the efficient degradation and resource utilization of target pollutants. This study aims to raise awareness of the use of photocatalytic microbial fuel cells for pollutant degradation by providing an overview of the practical status of photocatalytic microbial fuel cells. This is achieved by reviewing the key cathode development, production capacity, and progress in the degradation of pollutants in photocatalytic microbial fuel cells. The issues facing future developments are also discussed in terms of how photocatalytic microbial fuel cells work and how they degrade pollutants. This study shows that photocatalytic microbial fuel cells are beneficial for achieving renewable energy (bioenergy, photovoltaic, etc.) capacity and dealing with environmental pollution and that this is a novel technology that deserves to be promoted to achieve the current dual carbon targets.

## Introduction

Environmental issues are more closely linked to the development of human society than energy issues and are a direct threat to the health and survival of the present generation. Of all the environmental problems, the one that has the greatest impact on mankind is the pollution and destruction of the water environment. Therefore, wastewater treatment has become an extremely important element in the management of environmental problems. Wastewater treatment technology has evolved to include electrolysis (Li et al., 2014), incineration (Cai et al., 2020), membrane separation (Ahmad et al., 2021), deep well injection (Hongkun and Lin, 2007) and biological treatment (Velvizhi and Venkata Mohan, 2015; Kong et al., 2019). Each of these five treatment methods has its advantages and disadvantages: electrolysis is highly adaptable and can efficiently treat a wide range of wastewaters (G ao et al., 2006), with the disadvantage of higher costs (Hong, 2001); incineration is simple to implement and has a high treatment rate, with the disadvantage that the treatment effect is greatly influenced by the concentration of organic matter in the wastewater, and incineration itself is only suitable for treating wastewater with high concentrations of organic matter, while in treating wastewater with low concentrations of organic matter it is necessary to adjust the pH value of the wastewater, add fuel (Wei et al., 2008) and bring about combustion (Jiehong, 2010). The membrane separation method has the advantages of simple principle, easy operation, and no secondary pollution (Kim, 2011), but the disadvantage is that the membrane is easy to be clogged (Schofield et al., 1990) and the cost is high; the advantage of the deep well irrigation method is that it is easy to implement, but the disadvantage is that it will lead to secondary pollution (Shi et al., 2014); the biological treatment method has the advantages of high adaptability, a wide range of application, low cost and high efficiency (Abou-Elela et al., 2010), which is the current The main application method for wastewater treatment (Takata et al., 2013).

Environmental pollution is becoming increasingly serious and the search for clean treatment methods is urgent. Among them, photocatalysis has been widely noticed because of its thorough mineralization, catalyst stability, and mild reaction. In recent years, photocatalysis (PCO) has been used in the field of air purification, which can degrade air pollutants such as formaldehyde and volatile organic compounds into harmless end products such as CO_2_ or H_2_O and other small molecules at ambient temperature using light energy by using nanostructured photocatalytic materials with unique properties as catalysts, and without significant energy consumption. Photocatalysis is therefore considered to be a promising air purification technology and a promising green project on a global scale, contributing significantly to contemporary developments in global cleanliness, environmental management, energy, and related fields. The main problems faced in the practical application of photocatalysis are low quantum efficiency, low photocatalytic efficiency i.e. easy compounding of photogenerated electron-hole pairs, high cost, loading and separation, and recovery of photocatalysts. To improve the application of photocatalysis, researchers have researched catalysts from a range of perspectives such as doping extension, multiphase catalysis, and atomization, and through continuous efforts, photocatalysis is becoming more and more widely used. The current Frontier of innovation in photocatalysis is the coupling of other advanced oxidation techniques with photocatalysis to enhance the photocatalytic effect. From an energy point of view, pollutants store a large amount of chemical energy. To find a way to control environmental pollution while converting this chemical energy into energy that we can use, the photocatalytic fuel cell (PFC), designed based on the principle of the primary cell based on semiconductor photocatalytic oxidation technology, was created. Photocatalysis (PEC) is a method of introducing an electric field into photocatalysis, effectively inhibiting the recombination of electron-hole pairs and thus increasing the catalytic rate without creating secondary pollution. Photocatalytic materials are used as photoelectrodes and act as light absorbers and photocatalysts. The n-type semiconductor catalysts form the photoanode, which catalyzes only oxidation reactions, and the p-type semiconductor catalysts form the photocathode, which catalyzes only reduction reactions. It has been shown that lower voltages can accelerate the electron-hole separation and thus increase the efficiency of photocatalysis (Liu et al., 2011). Microbial fuel cell (MFC) technology can generate a certain amount of current while enabling the bacterial oxidation of pollutants (Liu et al., 2017). It has the advantage of being a win-win situation for both energy and the environment. Therefore, the synergistic effect of the catalytic oxidation system can be further enhanced if the MFC is used to provide the low voltage required for the PEC process, forming a photo catalytically coupled microbial fuel cell system (PMFC for short). The PMFC system is more effective in degrading pollutants (Wang et al., 2018), where both photovoltaic and bioenergy are utilized, and the conversion and removal of pollutants can be achieved simultaneously with the production of electricity through microbial metabolic processes. The system has been successfully applied to the degradation of various types of wastewater such as denitrification wastewater, coking wastewater, and antibiotic wastewater, using the synergistic effect of photovoltaic microorganisms. The sustainability of the system has received widespread attention from scholars and has broad prospects for development in the treatment of wastewater and renewable energy development (He et al., 2018; Antolini, 2019; Vasseghian et al., 2020; Mishra et al., 2021). A hierarchically photocatalytic microbial fuel cell system (PMFC) coupled with TiO_2_ photoanode and bioanode was established to enhance the power generation based on single-chamber MFC (Wang et al., 2022). Microbial fuel cell (MFC) has emerged as a promising technique, which can produce useful electrical energy from organic wastes and decontaminate polluted water (Suresh et al., 2022). The prospect of wastewater treatment technologies mostly emphasizes processing efficiency and economic benefits (Selihin and Tay, 2022). Photocatalytic oxidation has been widely investigated and applied to perform the degradation of organic pollutants in water and air. In recent technological advancements, photocatalysis (PC) is integrated into fuel cell (FC) to form photocatalytic fuel cell (PFC) for simultaneous wastewater treatment and production of electricity (He et al., 2022).

To use photocatalytic microbial fuel cell technology effectively, it is necessary to review current research advances and challenges. In this work, the potential of photocatalytic microbial fuel cells and applications for pollutant degradation are explored, and general guidelines for nanotechnology for cell cathode materials are discussed. The relationship between light conditions, cell devices, and microorganisms on photocatalytic microbial fuel cell productivity is investigated. Future directions are also envisaged based on the principles and research progress of PMFC degradation of pollutants.

## Composition and Working Principle of Photocatalytic Microbial Fuel Cells

### Composition of Photocatalytic Microbial Fuel Cells

**FIGURE 1:**
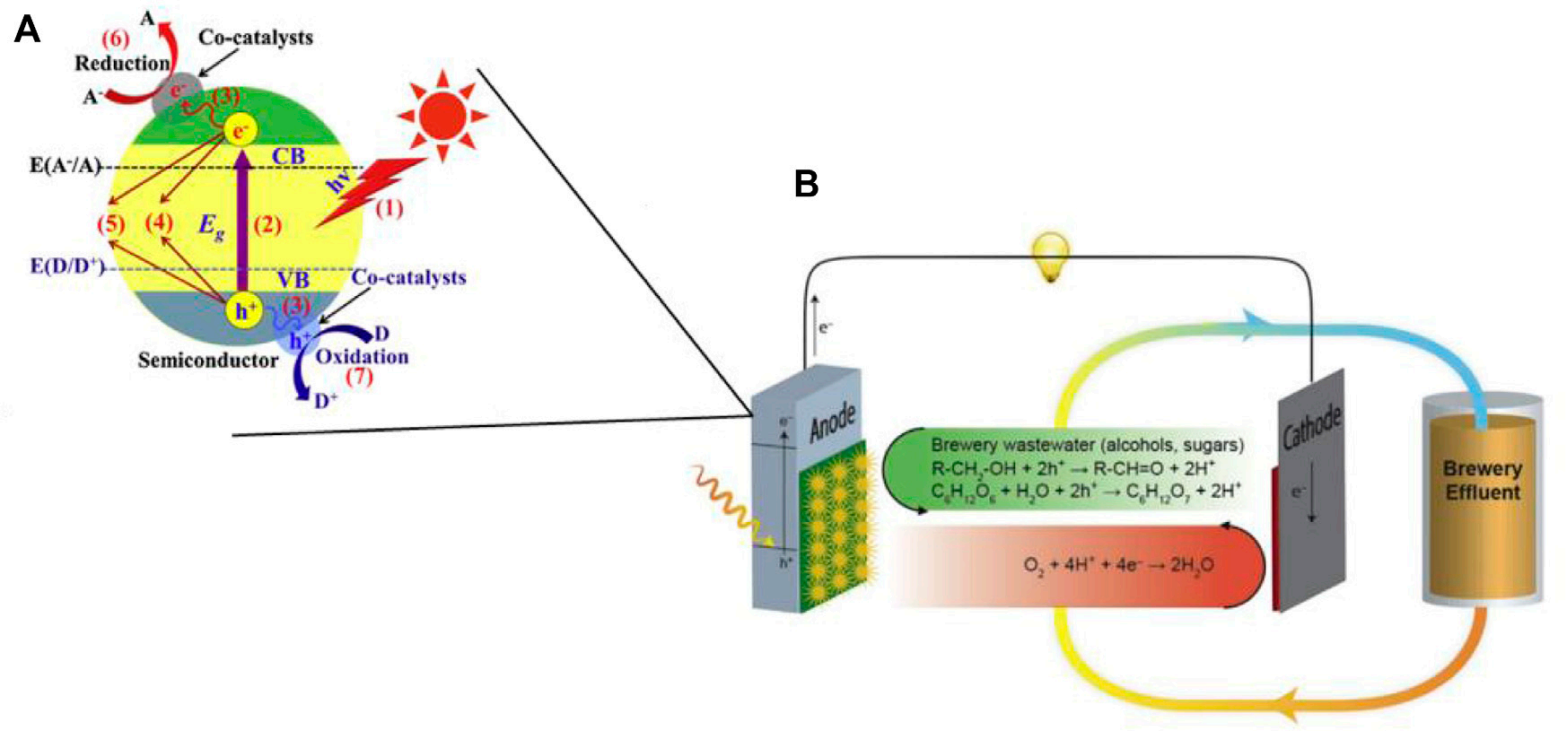
**(A)** Basic principle of semiconductor cathode photocatalysis (Wen et al., 2017); **(B)** Schematic diagram of photocatalytic microbial fuel cell principle (Lui et al., 2019).

The principle of operation of a photocatalytic microbial fuel cell is shown in Figure 1. A two-chamber structure is used as an example, consisting of an anode, a cathode, and two chambers separated by an ion-exchange membrane. The anode and cathode are connected by wires, and photogenerated electrons flow from the anode to the cathode to generate electrical energy. The cathode undergoes a reduction reaction and the cavity of the anode undergoes a series of oxidation reactions, thus completing the simultaneous control of pollution and production. The photocatalyst produces photogenerated electrons under light excitation, as shown in Figure 1A. Due to the potential difference between the cathode and anode, the photogenerated electrons are transferred to the cathode via an external circuit thus generating electrical energy, while the holes oxidize and degrade the pollutant, recovering the chemical energy therein (Lui et al., 2019) as shown in Figure 1B. As the transfer of photoelectrons reduces the compounding rate of photogenerated electrons and holes, the catalytic effect of the photocatalyst is enhanced. However, there are some limitations in the research development of photocatalytic fuel cells: strong dependence of the photoanode material on TiO2, poor visible light utilization, and low quantum efficiency. Although a wide variety of semiconductor composite photocatalytic electrodes have been developed, how to improve the quantum efficiency and increase the removal efficiency of pollutants is still a top priority to be explored (Li et al., 2019a).

### Principle of PMFC Degradation of Pollutants

**FIGURE 2:**
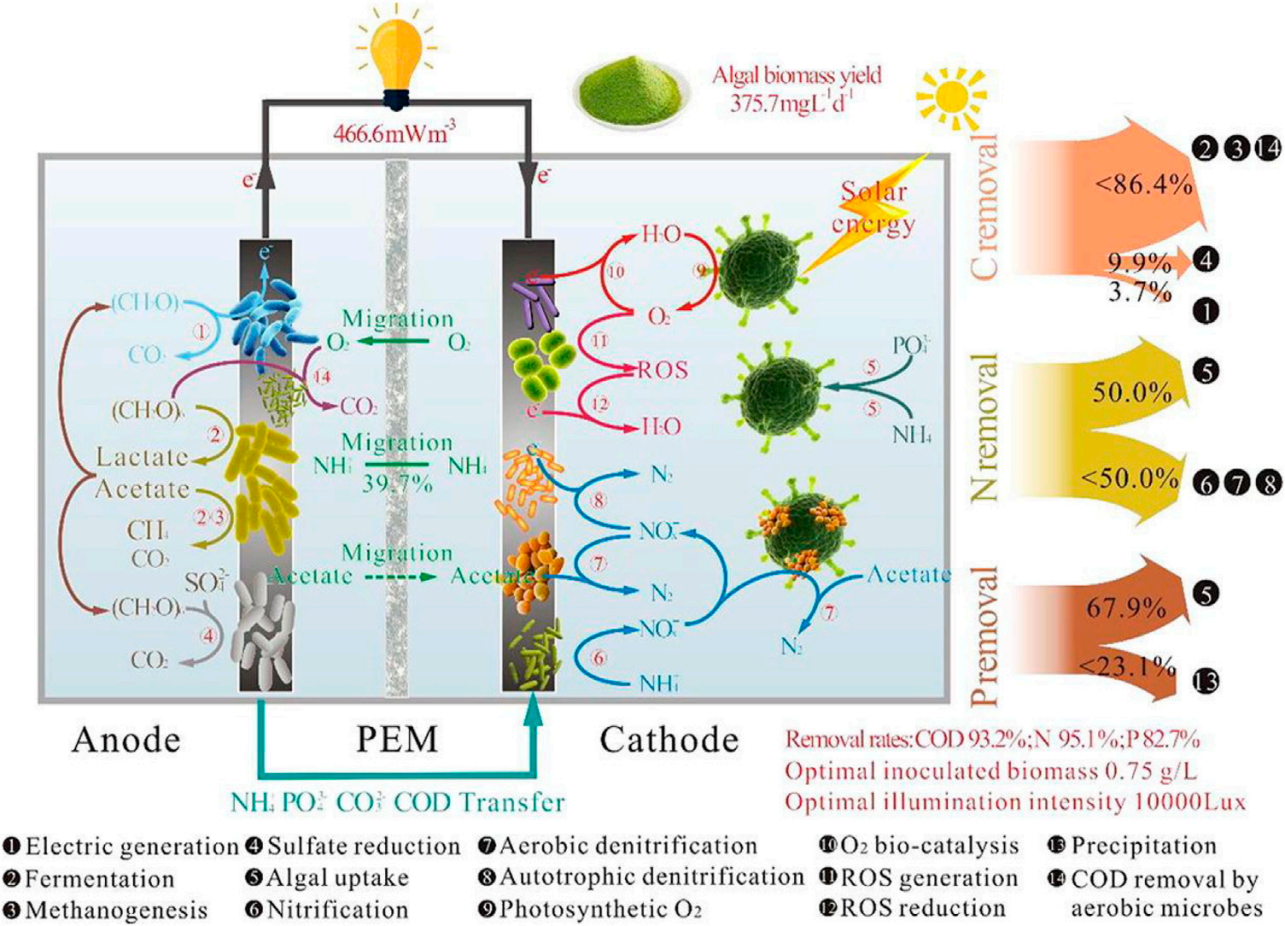
Principle of PMFC degradation of pollutants (Wang et al., 2019a).

Photocatalytic assisted microbial treatment technology is where photocatalysis and biodegradation take place simultaneously in the anode chamber. The photocatalytic material degrades the pollutant and the microorganism simultaneously under light illumination, and the two co-use the pollutant substrate through synergistic action, while the intermediate products produced during the degradation process of both can also be used as the substrate for each other. This method allows for better mineralization of organic pollutants and increases the rate of mineralization of organic pollutants, as shown in Figure 2. The pollutants are effectively removed by the synergistic action of the photocatalyst and microorganisms. The bio-anode is also connected in series with the photocatalytic anode, which can also be used to generate more electricity by combining the electricity production functions of both. The advantages of the two are fully combined. Initial treatment systems using photocatalysis and microorganisms to treat pollutants are sequentially coupled because they involve a two-step process of oxidation followed by biodegradation. In the ideal case of sequential coupling, controlled advanced oxidation converts the refractory organic compounds into biodegradable intermediates, followed by a biodegradation stage. The semiconductor energy band is composed of a valence band and a conduction band. When the photocatalyst on the anode receives light, the photoanode electrode with the nanostructured photocatalyst absorbs photons and generates electron-hole pairs, the electrons will leap from the valence band to the conduction band of the semiconductor, the holes on the valence band oxidize the substrate through their own strong oxidizing photogenerated holes or generated radicals such as hydroxyl radicals, ultimately oxidizing the pollutant through a chain reaction Oxidation to small inorganic molecules. In the presence of a potential difference between the cathode and anode, the electrons generated at the anode are transferred from the photoanode electrode via an external circuit to the cathode where they undergo a reduction reaction to reduce the electron acceptor. The protons then pass through the proton exchange membrane to the cathode. Photogenerated holes are consumed by oxidation reactions, such as the oxidation of water or the oxidation of water-soluble organic or inorganic substances. Both the photoanode and cathode electrodes are in contact with the electrolyte, in which organic fuels and various ionic substances are dissolved. However chemical conversions beyond this pre-assumed oxidation point usually waste oxidants and increase operating costs without further benefit. The use of visible light as an alternative to UV light has also become a hot topic of research in recent years to save energy (Zhang et al., 2019a; Zeng et al., 2019; Qian et al., 2020). Some studies have shown that visible light is also effective as a light source.

The strong oxidation characteristics of photocatalytic fuel cells determine their properties as simple operation, wide oxidation range, non-selectivity, high applicability, and low operating conditions. However, it also suffers from poor target selectivity, the oxidation of target organic matter while generating more by-products, or non-target oxides being oxidized and reacting with the target oxides to produce unknown toxic substances.Numerous experiments have also shown that many pollutants and wastewater can be degraded by using them as photocatalytic fuel cell substrates with high degradation efficiency while generating electricity. Most researchers have investigated methanol, ethanol, or glycerol as fuels because these substances are more productive; have been extensively studied and well documented; are products of biomass, and are therefore renewable. Moreover, glycerol is a by-product of biodiesel (Pagliaro et al., 2007). In addition to small chain length alcohols and glycerol, other polyols, sugars, organic acids, aldehydes, and ketones have become common choices for hydrogen production from PFC (Antoniadou and Lianos, 2009). Photocatalysts act as oxidants for substances with the general formula C_x_H_y_O_z_, decomposing and mineralizing with water in the absence of O_2_ (Patsoura et al., 2007).

## Research Progress of PMFC

### Photocatalytic Cathode Materials

The photovoltaic electrode, as a synergistic party with the bioelectrode, has to have a significant photocatalytic effect, as well as high utilization of sunlight. Thus, photocatalytic materials used as photoelectrodes need to respond well to visible light. With the continuous development of solar photocatalysis (Zou et al., 2001) and photoreactors (Rodrıguez et al., 2004), especially new photocatalysts synthesized and fabricated using nanotechnology, the photocatalytic efficiency of specific materials can be greatly improved (Spasiano et al., 2015). The coupling of microorganisms to light energy is accomplished by a redox reaction determined by the redox potential of the bacterial cells on the anode and the position of the semiconductor band edge of the photocathode. To receive electrons from microorganisms, the semiconductor should have a higher valence band edge than the electrochemical potential activity of the bacterial cell. For example, the reported OmcA values for the bacterial outer membrane proteins of Schwannia cells compared to standard hydrogen electrodes (NHE) are -0.32 to −0.24 V and MtrC values of ∼ −0.1 V. When a semiconductor is selected as the cathode to couple to the Schwannia bioanode, the semiconductor valence band edge potential should be higher relative to the bacterial outer membrane proteins, and the cathode surface electron acceptor depends primarily on the electrolyte composition within the cathode chamber and the oxidation potential of the cathode surface. With the composition of the cathode chamber and the redox potential on the cathode surface, oxygen is a good electron acceptor for the cathode due to its abundant sustainability and environmental cleanliness (Cheng and Logan, 2007). The coupling of the bioanode to the photocathode results in an internal bias in the MFC system, electrons from the anode migrate through the external circuit to combine with the photocathode holes, electrons from the photocathode are released, organic matter is degraded and water is reduced (Chen et al., 2012).

Currently known semiconductor crystal structures of the p-type are much less common than the n-type (e.g., TiO_2_, ZnO, Fe_2_O_3_, WO_3_). As one of the few metal oxides that naturally displays p-type conductivity, cuprous oxide (Cu_2_O) is considered to be the most promising material for efficient photovoltaics for the following reasons: 1) it has a bandgap of ∼2.0 eV, thus ensuring efficient visible light absorption; 2) it has a conduction band position of 0.7 eV, which is more negative than the hydrogen reduction potential, which allows Cu_2_O to act as a photocathode for the reduction of water (Jia et al., 2019); 3) there is a natural abundance of copper in the crust to ensure the scale of Cu_2_O production at the photocathode. However, two main factors limit the practical application of Cu_2_O in PEC systems: 1) the mismatch between the intrinsic Cu_2_O current subdiffusion depth (typically 20–100 nm) and the optical absorption depth near the bandgap (∼10 μm); 2) the lower stability due to self-photo corrosion in electrolyte solutions (Zhang et al., 2013). Cu_2_O, as a p-type semiconductor material, has an optimal 2.0–2.2 eV bandgap, it has proven to be an efficient photocathode (Zhou et al., 2011) with promising applications in sensors (Liu et al., 2012), photovoltaic devices (Aryal et al., 2010), optoelectronic systems (Li et al., 2012), etc. One-dimensional (1-D) nanostructures (e.g. nanowires, nanorods, nanotubes) with the ability to independently regulate the actual carrier diffusion length of a semiconductor concerning the depth of light absorption capability are emerging as a solution to the carrier diffusion length-light absorption depth mismatch that often occurs in many semiconductors (Barreca et al., 2011).Methods for the synthesis of one-dimensional Cu_2_O nanostructures have also recently emerged, such as Qian et al. (Qian et al., 2010) used chemical methods to synthesize one-dimensional Cu_2_O nanowire arrays in form, and Reisner et al. (Lin et al., 2012) produced Cu_2_O one-dimensional nanostructures by electrochemical anodic oxidation. On the other hand, it has been shown that Cu_2_O photo corrosion occurs at the Cu_2_O/electrolyte interface, and as a reasonable scenario, the semiconductor can be naturally covered with a protective layer blocking direct contact with the electrolyte solution (Paracchino et al., 2012).

**FIGURE 3:**
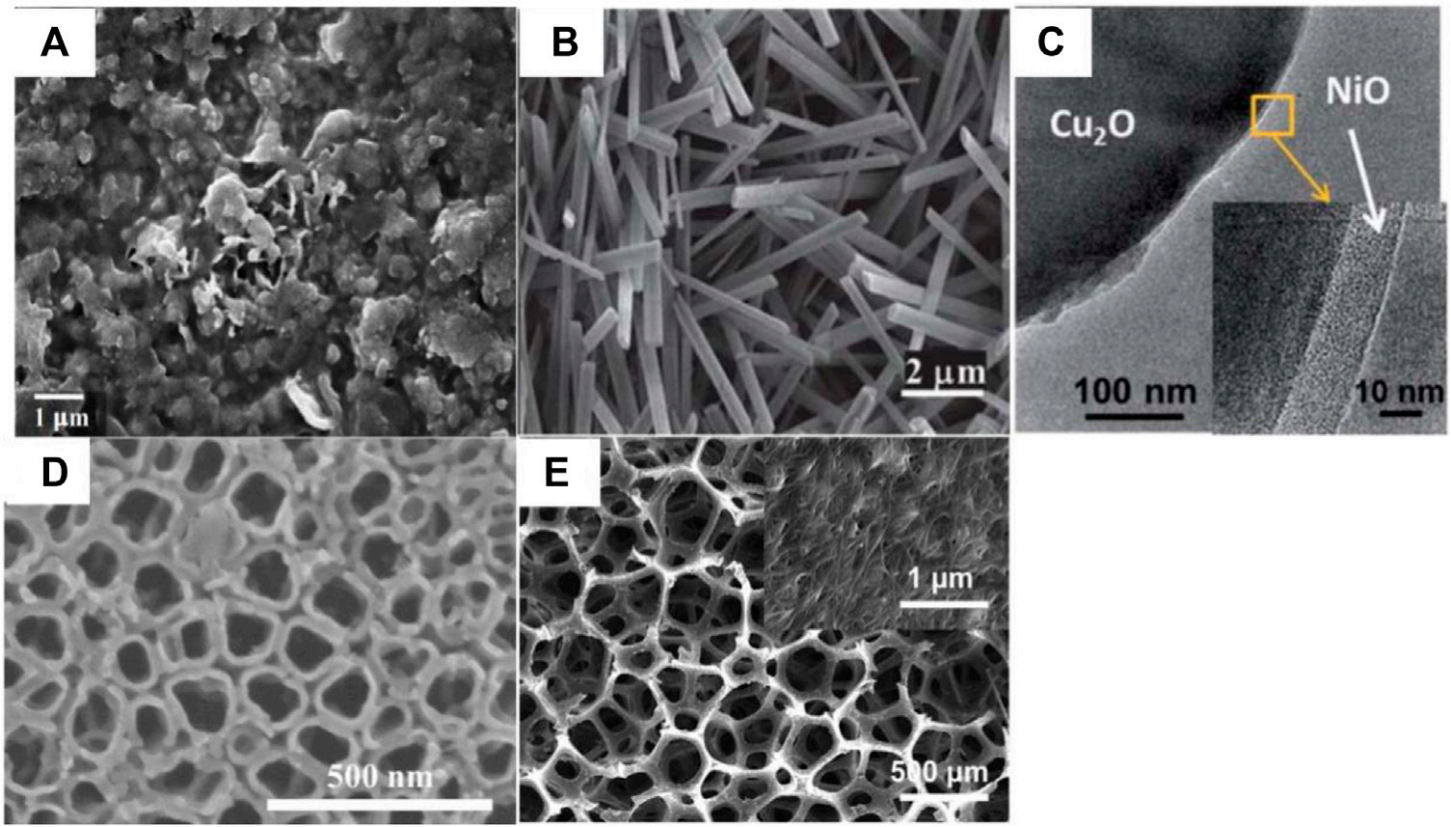
Common nanostructured semiconductor cathodes: **(A)** Cu_x_O electrodes (Castresana et al., 2019); **(B)** Cu|nanoCu(OH)_2_ and **(C)** Cu|nanoCu_2_O|NiOx (N = 3) electrode (Lin et al., 2012); **(D)** PDA/TiO_2_ NTs (composed of polydopamine modified TiO_2_ nanotubes) (Zeng et al., 2019); **(E)** Carbon nanotube (Xie et al., 2012).

Ordinary nanostructure semiconductor cathode material characterization in shown in the Figure 3. A summary of the current research on photocatalytic thin-film materials has implications for the preparation of photocatalytic cathodes in photocatalytic microbial fuel cells. The most widely used methods for the preparation of photocatalytic thin films are sol-gel, direct oxidation, hydrothermal and solvent thermal methods, and metal-organic chemical vapor deposition. The sol-gel method uses Ophthalmol salts as the titanium source, alcohol solvents such as ethanol as the organic medium, and inorganic acids and other catalysts to carry out hydrolytic polycondensation reactions to obtain colloidal dispersed TiO_2_ sols, and then uses the chosen substrate material to spray, lift, centrifuge and rotate the sol on the surface of the substrate and obtain TiO_2_ photocatalytic thin-film materials after high-temperature baking (Legrand-Buscema et al., 2002). However, this method is cumbersome, has a long preparation cycle, usually takes weeks or days, and has poor gel adhesion and high drying and sintering shrinkage, which can produce cracks and peeling on the substrate surface. Metal-organic chemical vapor deposition uses hydrogen to feed metal-organic vapors and gaseous non-metallic hydrides onto a heated substrate in a reaction chamber, which is then heated to decompose so that epitaxial layers can be grown on the substrate (Aburaya et al., 2001). This method requires high equipment, is expensive, and is less commonly used in the laboratory. Direct oxidation methods use strong oxidants or anodic oxidation to directly oxidize the corresponding metal to obtain semiconductor photocatalytic materials. Peng (Peng and Chen, 2004; Peng et al., 2005) used various oxidizing atmospheres such as oxygen, formic acid, water, ethanol, and acetone to directly oxidize titanium plates at high temperatures, resulting in rutile-phase TiO_2_ nanostructures of various morphologies on titanium plates. It was found that when oxygen was used as the oxidizing atmosphere, only crystalline films could be formed, whereas when acetone was used as the oxidizing atmosphere, neat and ordered arrays of TiO_2_ nanorods could be formed on the titanium surface. The hydrothermal and solvothermal methods involve the use of water or organic solvents as a medium to dissolve the precursors in the reaction system, forming atomic or molecular growth elements and finally nucleation crystals, in a closed reactor with a titanium source under high temperature and pressure. A matrix material with a similar lattice structure to that of the precursor is placed in the reactor to allow nucleation crystals to form naturally on the surface of the matrix.

Lu et al. (Lu et al., 2010) constructed a photocatalytic dual-chamber microbial fuel cell using natural rutile-coated graphite as the cathode and graphite plates as the anode, while inoculating the anode chamber with anaerobic sludge and using KCl as the electrolyte in the cathode chamber. To investigate the response of this type of microbial fuel cell to light, they compared the performance of the device in terms of electricity production under light and the absence of light. The device was placed in the absence of light for a long period to wait for the system to stabilize, and then exposed to a simulated natural light. After 50 min of steady irradiation, the output voltage stabilized, after which the light source was switched off and the voltage of the device dropped rapidly, returning to around 340 mV within approximately 50 min. The maximum power density of the device was 12.03 W/m^3^ in the light and 7.64 W/m^3^ in the absence of light, with an increase of 4.39 W/m^3^ in the light compared to the absence of light. Ding et al. (Ding et al., 2010) constructed a similar reaction device, but replaced the electrolyte in the cathode chamber with methyl orange (MO) and KCl. They designed a series of controlled experiments to analyze the effect of light and cathode material on the electricity production of photocatalytic microbial fuel cells with or without light, with or without rutile coating of the cathode, and with or without closure of the device. The maximum current density was found to be the highest in the closed microbial fuel cell system when the rutile-coated cathode was simultaneously illuminated, with a cathode current density of approximately 0.164 A/m^2^. Li et al. (Li et al., 2009) conducted similar experiments comparing the effect of the presence or absence of light on the potential generated by microbial fuel cells with clad rutile cathodes and found that the maximum potential generated was 0.80 V (for SCE) under light conditions and 0.55 V (for SCE) under light-free conditions. The experiments demonstrate that the use of rutile-coated cathode microbial fuel cells does have a relatively good response to light and can improve the cell’s electricity production performance under the light. Of course, in addition to the semiconductor cathode materials mentioned above, many new cathode materials have been developed (Ahmadpour et al., 2020; Xu et al., 2020a), but still, revolve around nanomaterials and composite semiconductor materials. The electrode material influences the electrical potential of the battery on the one hand and the capacity of the battery on the other. By choosing the right electrode material with a matched system and a reasonable nanostructure is the focus of future research.

### Capacity of Photocatalytic Microbial Fuel Cells

**FIGURE 4:**
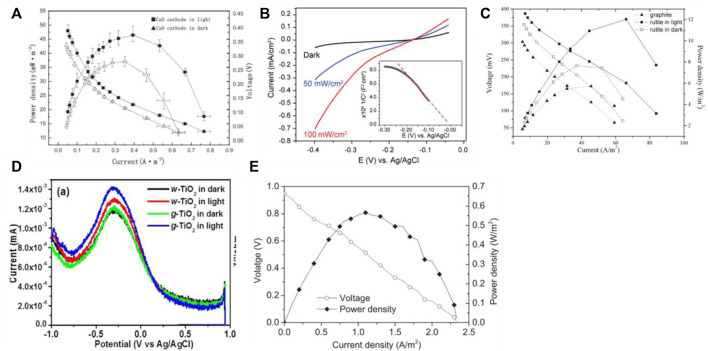
**(A)** Power density curves of PMFC with nanowire arrays of CuO as photocathode (Sun et al., 2015); **(B)** Power density curves of PMFC with Cu_2_O nanowire arrays as photocathode (Qian et al., 2010); **(C)** Power density curves of PMFC with rutile-coated graphite as photocathode (Lu et al., 2010); **(D)** DPV (differential pulse voltammetry) curves (Khan et al., 2018); **(E)** power density curves of PMFC with PANi@CNTs/SS as bioanode and CuInS_2_ as photocathode (Xu et al., 2020b).

The photocatalytic microbial fuel cell capacity is now of greater interest, such as the performance of electricity production and the rate of clean energy H_2_ production. Sun et al. (Sun et al., 2015) used a nanowire array of CuO as the photocathode and a carbon fiber brush inoculated with anaerobic sludge as the bioanode to construct a two-chamber H-type photocatalytic microbial fuel cell. The anode and cathode chambers were separated by a Cation Exchange Membrane (CEM), and the cathode chamber used a 0.5 mol/L Na_2_SO_4_ solution as the electrolyte and was aerated to maintain sufficient dissolved oxygen. As shown in Figure 4A, the cell performance was investigated in both dark and simulated natural light conditions, resulting in a power density of 46.44 mW/m^2^ in light conditions, an increase of 126% compared to 36.99 mW/m^2^ in dark conditions, and an open-circuit voltage of 466 mV in light conditions, an increase of 113% compared to 412 mV in dark conditions. The polarization curves showed that the internal resistance of the device was 333 Ω in the light, which was 6% lower than that of 354 Ω in the absence of light. The electrochemical impedance spectra showed that the solution resistance and electron transfer resistance of the photocathode CuO were 179.9 and 7.92 Ω, respectively, in the dark, and decreased to 138.6 and 2.04 Ω, respectively, in the light. Fang et al. (Qian et al., 2010) used p-type Cu_2_O in the form of nanowire arrays responsive to visible light as the photocathode and carbon cloth inoculated with an electrogenic bacterium (Shewanella oneidensis MR-1) as the bioanode to form a solar-driven photocatalytic microbial fuel cell. The anode and cathode chambers were separated by a CEM using a 0.1 mol/L potassium phosphate buffer solution as the electrolyte and a tryptic soy broth (TSB) as the substrate for the electrogenic bacteria in the anode chamber. The device was tested in the presence and absence of light. As shown in Figure 4B, the instantaneous photovoltage of the photocathode reached 0.25 V (relative to the Ag/AgCl electrode, below) at a light intensity of 100 mW/cm^2^; the bioanode maintained a stable voltage of -0.3 V in the presence and absence of light, demonstrating the photoresponsive effect of the Cu_2_O photocathode. To verify the performance of the device and the synergistic effect of the bioanode and photocathode, a series of control experiments were carried out at zero bias voltage and light intensity of 20 mW/cm^2^. The device was able to generate a weak photocurrent of 0.6 μA when a Pt anode - photocathode was used; when a bioanode - Pt cathode was used, it exhibited a background current of 20 μA with no photocurrent generation; while the photocatalytic microbial fuel cell composed of a bioanode - photocathode produced a large current of 200 μA (50 μA/cm^2^), successfully demonstrating the synergistic effect of the bioanode and photocathode. The device can generate a continuous current with a continuous substrate supply.

Lu et al. (Lu et al., 2010) constructed a photocatalytic two-chamber microbial fuel cell using rutile-coated graphite as a photocathode and a bioanode. The two chambers were separated by a CEM, and oxygen was reduced on the surface of the cathode chamber using 1 mol/L KCl as the electrolyte to exploit the visible light response of natural rutile. The study used graphite plates as the anode and the anode chamber was inoculated with anaerobic sludge. The performance of the device in terms of electricity production under light and without light was compared to observe the effect of the light response of this type of microbial fuel cell. After the system had stabilized in the absence of light, the output voltage of the device was increased from 337 to 384 mV by switching from the absence of light to simulated natural light conditions; the maximum power densities of 12.03 and 7.64 W/m^3^ were obtained in the light and absence of light conditions respectively, as shown in Figure 4C. The above results demonstrate that the device has a good photoresponse and that light can improve the power production performance of the cell. Khan (Khan et al., 2018) et al. used narrow bandgap and visible light-activated TiO_2_ nanoparticles as the photocathode and 0.2 mol/L Na_2_SO_4_ solutions as the electrolyte isolated anode. after the modification of TiO_2_ in the PMFC cathode, a voltage of ∼0.3979 V was obtained, which was higher than the original 0.0465 V, and the power density was also increased from 54.88 mW/m^2^ to 70.39 mW/m^2^, as shown in Figure 4D. This is due to the enhanced photoelectrochemical response under visible light irradiation as the electron transfer resistance decreases and the charge transfer rate increases, further supporting the improved performance of g^−^ TiO_2_. This study also confirms that narrow bandgap TiO_2_ can be easily obtained and effectively used as a photocatalyst and photovoltaic material. Not only does the photocathode material have a significant impact on cell capacity, but also the anode (Wang et al., 2019b) and biocatalyst (Sarmin et al., 2021). Xu (Xu et al., 2020b) et al. used PANi@CNTs/SS as the bioanode and CuInS_2_ as the photocathode to construct a dual-chamber photocatalytic microbial fuel cell. OCV was 950mV, an 18.75% improvement. By adjusting the external resistance, the maximum power density was 0.566 W/m^2^, corresponding to a current density of 1.11 A/m^2^ and a voltage of 0.51 V, as shown in Figure 4E.

To investigate whether photocatalysis and microbial fuel cells have a synergistic effect, Li et al. (Li et al., 2009) conducted a comparative experiment with and without microbial inoculation in the anode chamber under the same conditions. It was found that the maximum output current obtained in the anode chamber inoculated with microorganisms was 0.125 mA, while the maximum output current obtained in the anode chamber without microorganisms was 0.09 m A. This demonstrates that photocatalysis and microorganisms do have a synergistic effect in terms of electricity production. The potential of the microbial fuel cell without photocatalysis is low after seven major potential losses, including the potential loss of the anode chamber electrolyte, the potential loss of the anode surface reaction, the potential loss of the anode electrode, the potential loss of the external resistance, the potential loss of the cathode electrolyte, the potential loss of the cathode surface reaction, and the potential loss of the cathode electrode, while the microbial fuel cell with photocatalysis gains potential in its cathode The photocatalytic microbial fuel cell gains an additional potential boost from the response of the photocatalytic material to light, which in turn increases the maximum potential of the microbial fuel cell (Legrand-Buscema et al., 2002).

The output power of a microbial fuel cell $\text{P}={\text{E}}^{2}/\text{R}$ , is mainly determined by two main factors: potential and internal resistance. A similar experiment conducted by Ding et al. (Ding et al., 2010) also found that the internal resistance of the device under light conditions was 443.4 Ω, which was 934.6 Ω lower than the device’s internal resistance of 1,378 Ω under no-light conditions. it can be seen that the internal resistance of a cell affects the capacity of the cell, and how to improve the power density and capacity efficiency is something that needs to be explored in the future. On the one hand, the electrode materials need to be optimized, and on the other hand, the cell system needs to be optimized to improve the matching between the electrode materials.

### Degradation of Pollutants by Photocatalytic Microbial Fuel Cells

**FIGURE 5:**
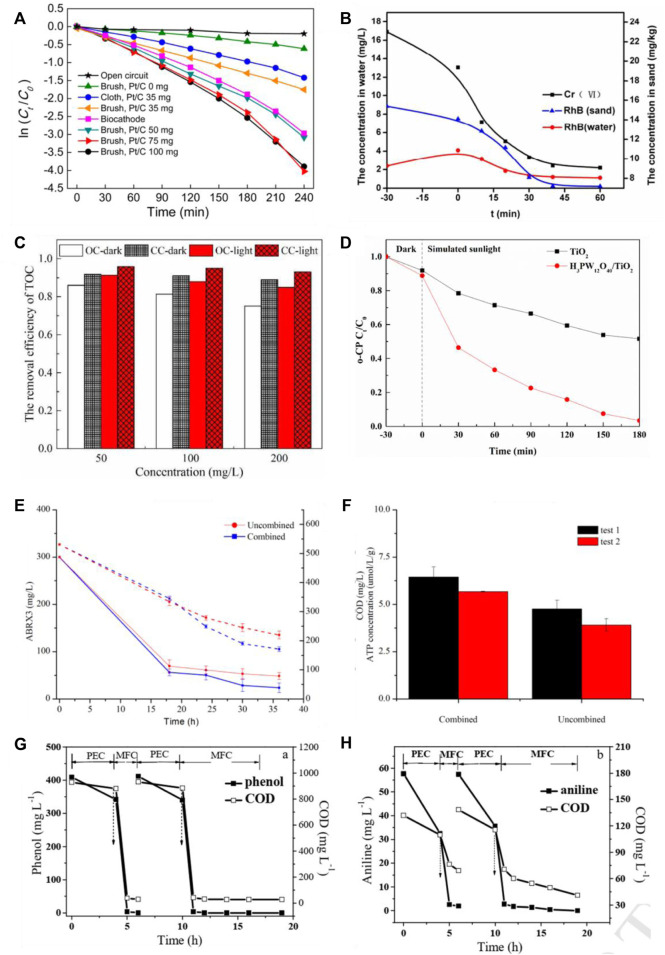
Effectiveness of photocatalytic microbial fuel cells for the degradation of pollutants: **(A)** removal of methyl orange (Du et al., 2014); **(B)** removal of Cr(VI) and RhB (in water and sand) (He et al., 2018); **(C)** removal of 2,4,6-trichlorophenol (2,4,6-TCP) at different concentrations (Wang et al., 2019b); **(D)** removal of chlorophenols (Li et al., 2019b); **(E)** removal of azo dyes and **(F)** COD (Long et al., 2017); **(G)** Phenol and **(H)** Aniline removal (Zhang et al., 2019b).

There are a few examples of photocatalytic microbial fuel cells used to remove pollutants. Li et al. (Li et al., 2009) investigated the removal of Cr^6+^-containing pollutants by a rutile-coated graphite photocathode in a photocatalytic microbial fuel cell. Under illumination, Cr^6+^ at an initial mass concentration of 26 mg/L was reduced by 88% after 22 h, an increase of 23% compared to the reduction rate in the absence of light. Du et al. (Du et al., 2014) investigated the degradation of methyl orange within the anode chamber using a photoelectric anode - biocathode composition with the same photoelectric anode configuration. The cathode was a carbon brush device loaded with different amounts of catalyst (Pt/C) compared to an open circuit + Pt/C (0 mg) carbon brush cathode, a closed-circuit + Pt/C (0 mg) carbon brush cathode, a closed-circuit + Pt/C (35 mg) carbon brush cathode, a closed-circuit + Pt/C (50 mg) carbon brush cathode, a closed-circuit + Pt/C (75 mg) carbon brush cathode, a closed-circuit + Pt/C (75 mg) carbon brush cathode, and a The degradation rate constants k for methyl orange were 0.0009-1, 0.0025-1, 0.0070-1, 0.0012-1, 0.0160-1, and 0.0012 min-1 for closed-circuit + biocathode, respectively, as shown in Figure 5A. The results showed that the open circuit methyl orange degradation rate was significantly lower than that of the closed circuit, and the closed-circuit + biocathode methyl orange degradation rate was comparable to that of the closed-circuit + Pt/C (50 mg) carbon brush cathode, demonstrating that the photoelectric anode and biocathode can produce synergistic effects. He (He et al., 2018) et al. investigated the effects of photocatalytic fuel cells and coupling of photocatalytic and microbial fuel cells on the removal of rhodamine B, antibiotics, and heavy metal ion contaminated wastewater, as shown in Figure 5B. In the PEC-MFC, the photocatalytic electrode was integrated with the bioanode and the Cr(VI) in the cathode chamber was rapidly reduced, as was the concentration of RhB in the sand, almost within 2 h. Cr(VI) in the sand was reduced within a few hours. Both systems were found to be successful in purifying contaminated wastewater and were found to be a cost-effective and sustainable method. the concentration of 200 mg/L, 79.3% were removed within 10 h, which was higher than the unirradiated MFC (66.0%) and the photocatalytic-only process (56.1%), and the current generated in the coupled system was higher than the other two systems (Wang et al., 2019b), as shown in Figure 5C. The addition of an external voltage of 0.5 V can cut the compounding rate of the photogenerated carriers of TiO_2_ and improve the degradation efficiency of chlorophenols (Li et al., 2019b), as shown in Figure 5D. However, the external voltage input requires a large amount of energy, which is not energy efficient and not conducive to practical applications.

Hou (Zhu et al., 2021) et al. presented a photocatalytic microbial fuel cell system with a novel photocatalytic air cathode for the synergistic degradation of 2,4,6-trichlorophenol (TCP) and simultaneous energy production. In addition, 50 mg L-1 TCP was completely degraded within 72 h with a degradation rate 1.8 times higher than that of the air-cathode MFC without N-TiO_2_ photocatalyst. Using the MFC-powered PC-MFC system, Long et al. (Long et al., 2017) achieved 97% degradation of azo dyes and 81% COD. Zhang et al. used Bi_2_MO_6_ as a photocatalyst to construct a PC system and indirectly coupled the MFC system to remove almost all phenol and aniline after 4 h of PC treatment followed by 2 h of MFC treatment (Zhang et al., 2019b), as shown in Figures 5G,H. In the *in situ* photoanodic PC-MFC coupled system, the catalytic material on the electrode surface is excited by light to release active radicals, which can degrade the hard-to-degrade pollutants into bioavailable substances that can be further decomposed and used by microorganisms. It was shown that the *in-situ* photoanode PC-MFC coupled system constructed by TiO_2_/Fe_2_O_3_ and g-C_3_N_4_ modified anodes removed 90.9% of hexavalent chromium (initial concentration 50 mg-L-1) and 79.3% of TCP (initial concentration 200 mg-L-1) (Ren et al., 2018). However, *in situ* photoanodic PC-MFC coupled system, higher demands are placed on the biocompatibility of the electrode materials and the environmental resistance of microorganisms. From the above, it is clear that the current photocatalytic microbial fuel cells have good removal and degradation effects on both wastewater containing heavy metals and wastewater containing pharmaceuticals, but due to the lack of current research data, the types and concentrations of antibiotics need to be expanded. At the same time, the system needs to be improved and the presumed degradation pathways need to be calibrated to move from the laboratory to practical applications.

## Summary and Outlook

**FIGURE 6:**
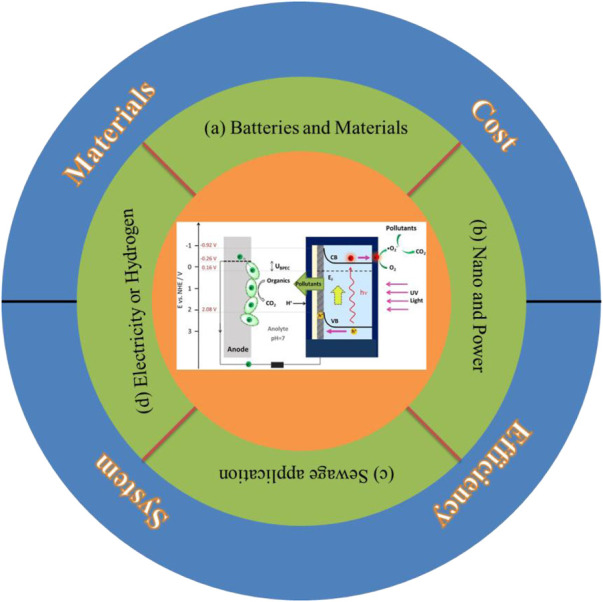
Research hotspots for photocatalytic microbial fuel cells.

Photocatalytic microbial fuel cells have good prospects for development as they can be applied to energy production and pollutant removal by utilizing the synergistic effect of photoelectrodes and bioelectrodes, which can simultaneously utilize solar energy and improve the productivity efficiency of microbial fuel cells. Currently, applications of photocatalytic microbial fuel cell systems are focused on the expansion of high-efficiency photoanode assemblies, binary photoelectrode fuel cells, and multi-purpose fuel cells, as shown in Figure 6. To improve the performance and economic viability of these systems, breakthroughs are still needed to address some key issues, such as.

(a) What is the correlation between the physicochemical properties of electrode materials and cell performance;
(b) How to incorporate state-of-the-art nanotechnology to further increase power production;
(c) Whether photocatalytic microbial fuel cell devices can be applied to real wastewater systems;
(d) How it can be effectively used for power or hydrogen generation.

The above-mentioned significant concerns are the focus of this review. The methods for preparing nanomaterials for cathode semiconductors are summarized. The capability of photocatalytic microbial fuel cells is summarized and explored in relation to pollutant degradation in photocatalytic microbial fuel cells. From the layout to the anode material and media composition, the anode chamber must be tuned as a microbial growth habitat. The device’s setup should reduce the reactor’s internal resistance while increasing power output.Although microbial fuel cells are very simple to build, the cathode, anode, diaphragm, and microbial fuel cell layout all have a substantial influence on the microbial fuel cell’s performance. Big electrode spacing, large electrode chamber size, limited effective volume of electrode chamber power output, and intermittent operation are all intrinsic problems with h-type microbial fuel cells. These drawbacks have a negative impact on the photocatalytic-microbial fuel cell’s performance and pollution removal. Furthermore, the photocatalytic cathode electrode in the cathode chamber’s center is not favorable to light absorption and solar irradiation via the electrode chamber and solution to the photoelectric electrode.Although using a photocatalytic anode to shield the carbon brush anode can prevent direct UV light from reaching the functional microorganisms on the carbon fiber brushes, resulting in their death, this approach cannot be completely avoided, and some UV light will still reach the carbon brush bioanode. More upgrades to the reactor’s construction are required. It is critical to construct low-cost and reliable devices in order to progress the practical use of photocatalytic MFC technology. To maximize the efficiency of solar light use, photovoltaic electrodes should be conveniently produced and low-cost semiconductor materials with a good bandgap. Furthermore, the photovoltaic electrodes in solution should be chemically stable.The advancement of photocatalytic microbial fuel cell applications is dependent on the development of microbial electrochemistry, semiconductor photocatalytic materials, microbial fuel cell configurations, and other technologies, and in-depth research on all aspects will aid in achieving renewable energy (bioenergy, light energy, etc.) production capacity and addressing pollution issues.
